# Supplementary figures and images for: Integrated Genomic Analysis of Primary Prostate Tumor Foci and Corresponding Lymph Node Metastases Identifies Mutations and Pathways Associated with Metastasis
Source: Cancers (Basel). 2023 Nov 30;15(23):5671. doi: 10.3390/cancers15235671 (PMC10705102; doi:10.3390/cancers15235671)

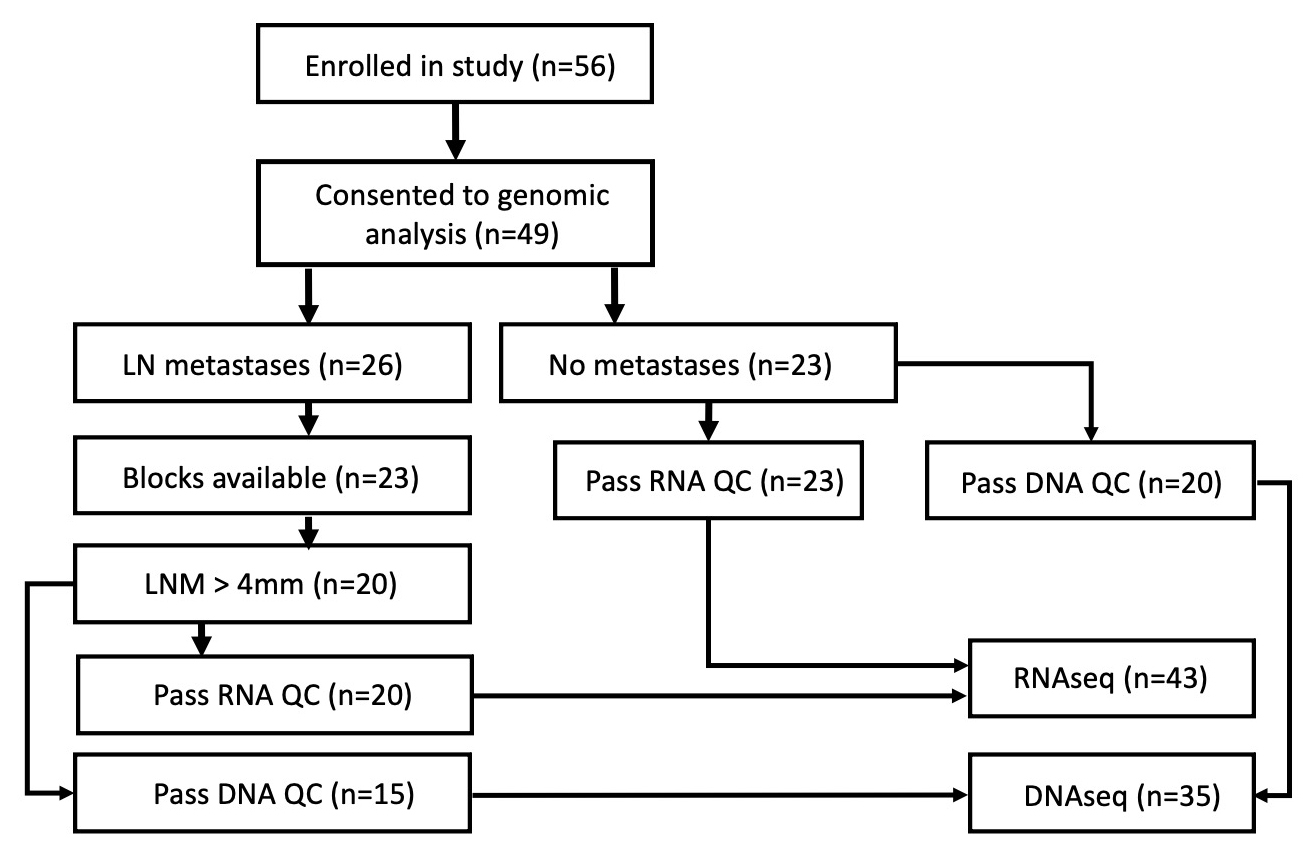

Supplement: Supplementary file 1 [file cancers-15-05671-s001.zip › Fig_S1_patient_sample_flowchart.jpg]

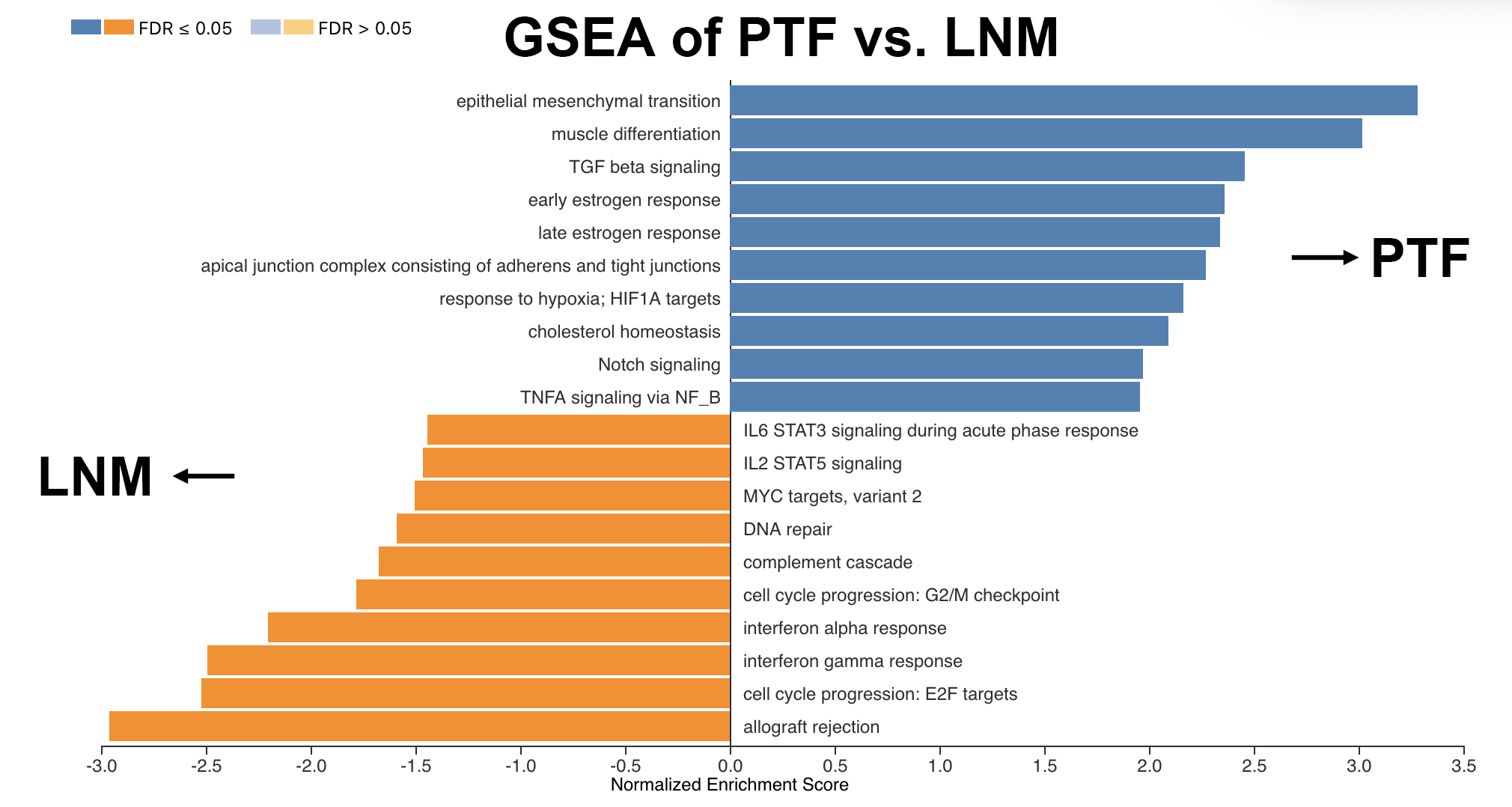

Supplement: Supplementary file 1 [file cancers-15-05671-s001.zip › Fig_S2_GSEA_PTF_vs_LNM.jpg]

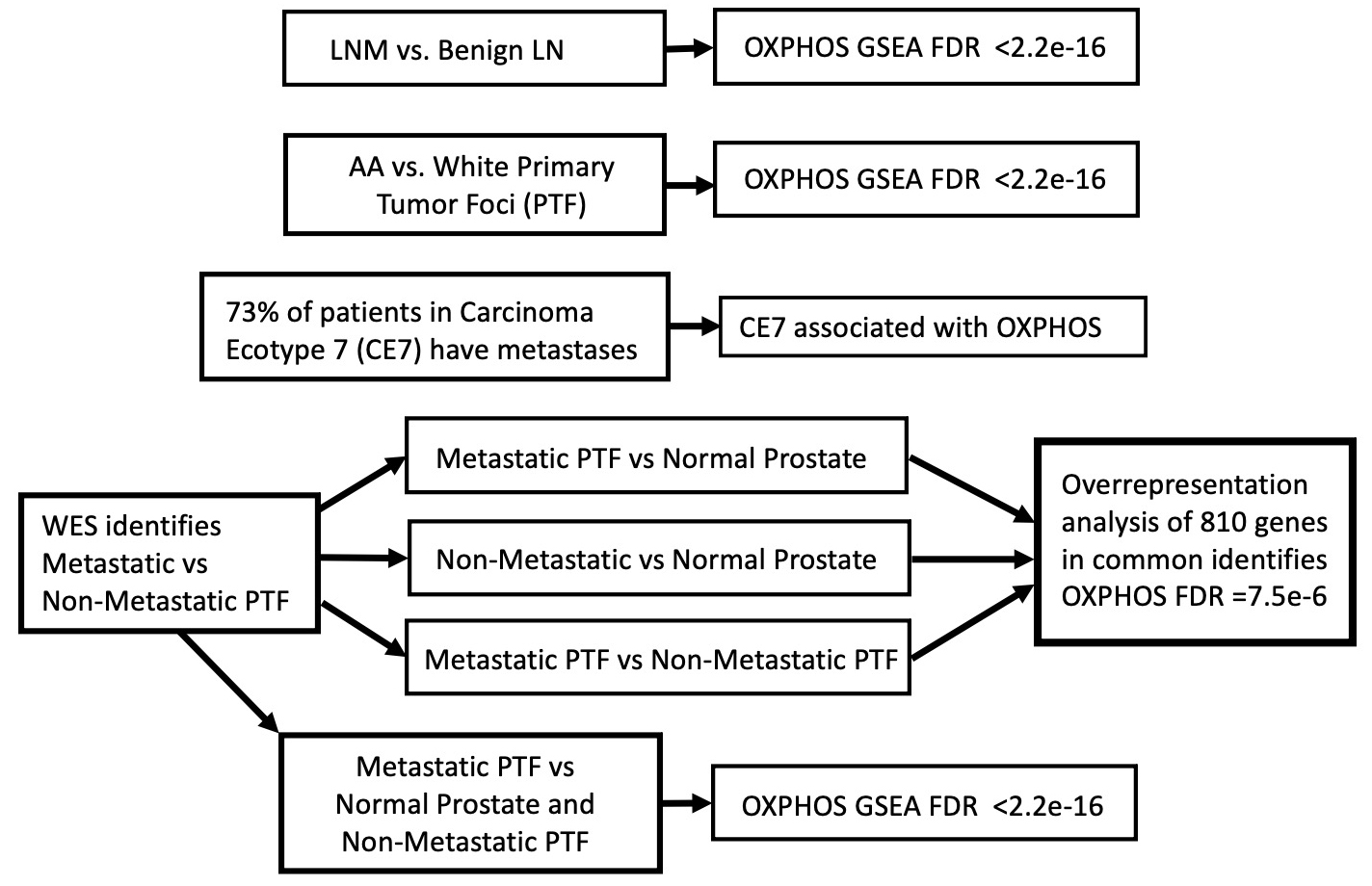

Supplement: Supplementary file 1 [file cancers-15-05671-s001.zip › Fig_S3_OXPHOS_geneset_flowchart.jpg]
